# Long-term outcome of juvenile dermatomyositis associated with lipodystrophy: experience of a university hospital^☆^

**DOI:** 10.1016/j.abd.2025.501130

**Published:** 2025-06-18

**Authors:** Igor Kapetanović, Mirjana Gajić-Veljić, Branka Bonači-Nikolić, Miloš Nikolić

**Affiliations:** a Clinic of Dermatology and Venereology, University Clinical Center of Serbia, Belgrade, Serbia; b Clinic of Allergy and Immunology, University Clinical Center of Serbia, Belgrade, Serbia; c University of Belgrade School of Medicine, Belgrade, Serbia

**Keywords:** Dermatomyositis, Lipodystrophy, Therapeutics

## Abstract

**Background:**

Juvenile Dermatomyositis (JDM) is a rare myopathy. Lipodystrophy is an under-reported chronic JDM complication.

**Objective:**

Assess the long-term outcome of JDM associated with lipodystrophy.

**Methods:**

Retrospective study of JDM patients who developed lipodystrophy, diagnosed and treated between 1^st^ January 1990 and 31^st^ December 2023, in a University Clinic of Dermatology. The mean follow-up was 150.8-months (range 29‒291).

**Results:**

Lipodystrophy was diagnosed in 5 children (4 girls and one boy, aged 3‒14 years) of 22 JDM patients (17 girls and 5 boys). Four patients had partial, and one had focal lipodystrophy that occurred 34.2-months (mean period) after the initial JDM symptoms. All five children had antinuclear antibodies, but none had dermatomyositis-specific/associated antibodies. No malignancies or visceral involvement were found. At JDM presentation, all 5 patients had low serum vitamin D, body mass index (BMI) ranged from 14.3 to 20.7. Triglycerides and fasting glucose levels were normal in all patients, while cholesterol was elevated in one patient. Despite Standard Immunosuppressive Therapy (IST), 2/4 of patients with initially partial lipodystrophy progressed to generalized lipodystrophy and 4/5 patients developed calcinosis 46 months (mean period) after JDM diagnosis. At the last check-up, 3/5 patients had chronic JDM course, requiring IST, while 2/5 patients were in remission, without IST, with only residual lipodystrophy.

**Study limitations:**

Retrospective study.

**Conclusions:**

During the 34-year period, 5/22 (23%) JDM patients developed lipodystrophy. Metabolic complications were not found. All patients had significant delays in JDM diagnosis. Early diagnosis and IST are necessary to reduce the risk of lipodystrophy, as a severe, chronic JDM complication.

## Introduction

Dermatomyositis (DM) is a rare, chronic connective tissue disease affecting primarily skin and muscles. DM is of unknown etiology, presenting as adult and juvenile disease (onset before 18). Juvenile Dermatomyositis (JDM) is the most common pediatric myopathy with an estimated incidence of approximately 3.1 children per one million.^1^ Proximal muscle weakness and characteristic cutaneous lesions are essential clinical manifestations along with signs of proximal extensor inflammatory myopathy. Before the glucocorticoid era, JDM led to death in one-third of patients, but with therapy and diagnostics advancing, mortality decreased to a currently reported 2%‒3.1%.^2^, ^3^, ^4^ While many achieve remission, long-term complications and specific damage may still develop such as lipodystrophy, calcinosis, residual weakness, joint contractures, muscle atrophy, hirsutism, growth failure, and dysphagia.^4^, ^5^

Lipodystrophy is a less frequent but significant complication of JDM. Lipodystrophy can be localized or generalized. Lipodystrophy can be acquired or congenital and may be associated with metabolic abnormalities such as hyperlipidemia, impaired glucose tolerance, insulin-resistant diabetes mellitus, acanthosis nigricans, and hepatomegaly.^6^, ^7^ Acquired Lipodystrophy (ALD) has been associated with autoimmune diseases.^7^ Childhood ALD has been linked to JDM,^7^ Systemic Lupus Erythematosus (SLE),^8^ panniculitis,^9^ Sjögren syndrome and juvenile rheumatoid arthritis.^10^, ^11^ Interestingly, lipodystrophy is associated almost exclusively with JDM although there have been published case reports of lipodystrophy affecting adult-onset dermatomyositis.^12^, ^13^, ^14^, ^15^, ^16^ The prevalence of lipodystrophy in JDM patients varies, ranging from 7.93% to 40%.^4^, ^5^, ^7^, ^12^^,^^17^, ^18^, ^19^, ^20^, ^21^ Lipodystrophy in JDM can be categorized as three phenotypes depending on the distribution of subcutaneous fat loss: generalized (loss in the face, trunk, abdomen, and all extremities), partial (upper and/or lower extremities, with relative sparing of abdomen and trunk), or focal (localized areas, resulting in skin surface depression or dimpling).^6^, ^22^, ^23^

In this report, we present four girls and one boy with JDM who developed different phenotypes of lipodystrophy. Clinical manifestations of JDM and associated lipodystrophy, laboratory analyses, complications, and treatment options during long follow-up were described.

## Materials and methods

From the database, we conducted a retrospective review and clinical data analysis of confirmed JDM patients associated with lipodystrophy at the Pediatric Dermatology Department of the University Clinical center of Serbia between January 1^st^ 1990 and December 31^th^ 2023. A database search of PudMed and Medline was undertaken using the keywords “lipodystrophy” OR “lipoatrophy” AND “juvenile dermatomyositis”. A reverse search of citations and additional cases was identified.

## Results

22 patients with JDM (17 girls, 5 boys – 3.4:1 female-to-male ratio) were diagnosed at and/or referred to our department. Cutaneous rash was seen in all the JDM patients (22/22) as the most frequent complaint at initial presentation, followed by arthralgia, myalgia, and weakness alone which were seen in 54.5% of patients (12/22) respectively, while 36.4% had a preceding infection (8/22). During a 34-year time frame, 477 adult DM patients (351 female, 126 male – 2.79:1.0 female-to-male ratio) were diagnosed. JDM represented roughly 4.2% of all DM cases and 26.5 times less frequent in our experience.

Lipodystrophy was found in 22.7% of JDM patients (5/22) (4 girls and one boy, aged 3‒14 years). The duration between symptoms and diagnosis of JDM was 8.8-months (range 2‒20). Lipodystrophy occurred 34.2-months (mean period) after the initial JDM symptoms. The mean follow-up period was 150.8-months (range 29‒291). According to the European League against Rheumatism and the American College of Rheumatology (EULAR/ACR) classification criteria for adult and juvenile idiopathic inflammatory myopathies and their major subgroups, patients had an idiopathic inflammatory myopathies (IMM) score of 10.4, 12.1, 11.2, 12.1, and 11.3 respectively and were categorized as definite.^24^ All patients were classified as chronic continuous. Specific internal organ involvement and malignancy were not detected during long follow-up. All five patients had normal triglycerides, High-Density Lipoprotein (HDL), Low-Density Lipoprotein (LDL), and fasting glucose levels at the presentation of lipodystrophy. Only 1/5 patients had elevated holesterol 6.38 (n.v. < 5.20 mmoL/L). No one patient had acantosis nigricans at presentation and during follow-up. All children had Antinuclear Antibodies (ANA) (Table 1), but none had Myositis-Specific (MSA) (anti-Jo, anti PL-7, anti PL-12, anti Mi-2 and anti-SRP) and associated (anti-RNP, anti PM-Scl70, anti PM-Scl-100, anti-Ku, anti-SSA/Ro 52) antibodies. No patient had low C3 and/or C4 levels of complement. 4/5 patients developed calcinosis after JDM diagnosis (mean period 46 months, range period 18‒72-months). All the patients had low serum levels of vitamin D at the presentation of JDM. At the presentation and during follow-up all patients had normal electrocardiogram, ultrasound of the abdomen and heart, chest X-Ray, and pulmonary function tests.

**Table 1:** Clinical presentation and long-term follow-up of five patients with JDM and lipodystrophy.

	Patient 1	Patient 2	Patient 3	a Patient 4	Patient 5
Age at diagnosis years and sex	14, F	8, F	8, F	6, F	3, M
Duration of JDM symptoms at diagnosis (months)	6	2	20	10	6
Follow-up, months (years)	185 (15.4)	179 (14.9)	70 (5.8)	29 (2.4)	291 (24.2)
Associated diseases	Celiac disease	None	Constitutio atopica	None	None
Disease course, last check-up	Only residual lipodystrophy, no therapy	Only residual lipdystrophy, no therapy	Chronic continuous low dose IST	Chronic continuous low dose IST	Chronic continuous. Does not take IST regularly
***Lipodystrophy and calcinosis***					
***Lipodystrophy***					
Distribution, last check-up	Generalized	Focal	Generalized	Partial	Partial
Location last check-up	Zygomatic, trunk, gluteus, thighs, upper extremities	Unilateral (left) cheek	Lateral aspects of face, proximal upper extremeties, lumbosacral region, gluteus	Bilateral proximal upper extremeties	Shoulder girdle/hands
From JDM symptoms months	21	20	21	6	103
From JDM diagnosis months	15	18	1	0	97
Progression	+ (from partial)		+ (from partial)	–	–
***Calcinosis cutis***	–	+	+	+	+
From JDM diagnosis, months	–	37	57	18	72
***JDM at diagnosis***					
IIM score^a^	10.4	12.1	11.2	12.1	11.3
Muscle/joint contractures	–	–	+	+	+
Facial erythema	+	+	+	+	+
Heliotrope sign and edema	+	+	+	+	+
Gottron’s papules or sign	+	+	+	+	+
V-sign	+	–	–	–	–
Shawl sign	–	–	–	–	–
***BMI, hypertrichosis***					
BMI, at diagnosis	20.3	15.8	14.3	15.8	20.7
BMI, last check-up	17.0	19	17.9	15.8	27.5
Hypertrichosis, at diagnosis	–	–	–	–	–
Hypertrichosis, last check-up	–	–	–	+	+
***Laboratory results and workup at the presentation***					
ANA, HEp-2 cells Nucleoplasm, L/titer	Homogenous 160	Homogenous 160	Homogenous 320	Homogenous 640	Course speckled 80
C3 (0.7‒1.73 g/L)	0.8	0.9	1.210	1.1	0.81
C4 (0.12‒0.36 g/L)	0.18	0.24	0.18	0.2	0.24
CK (0‒154 U/L)	↑ 444	↑ 783	90	90	↑ 759
LDH (F < 580, M < 760)	↑1691	↑ 859	↑ 1261	↑ 1261	423
Vit D3 (75‒210 nmoL/L)	↓26.4	↓25.8	↓40.5	↓ 36.3	↓11.2
EMNG	Mild myopathy	Mild myopathy	Normal	Normal	Mild myopathy

F, Female; M, Male; ANA, Antinuclear Antibody; JDM, Juvenile Dermatomyositis; CK, Creatine Kinase; LDH, Lactate Dehydrogenase; ECG, Electrocardiogram; EMNG, Electromyoneurography; U/S, Ultrasound. IIM, Idiopathic Inflammatory Myopathies; IST, Immunosupressive Therapy; BMI, Body Mass Index..

a EULAR/ACR classification criteria for adults and juvenile IIM (definite >7.5 without muscle biopsy).

### Patient 1

**Figure 1:**
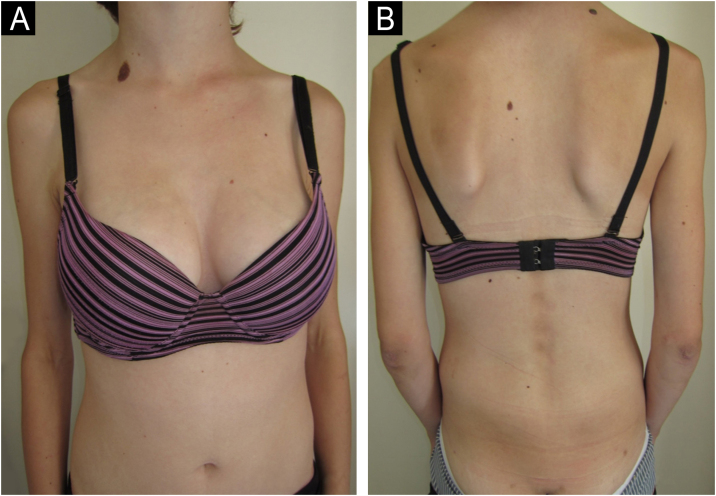
(A‒B) Patient nº 1 with progressive generalized lipodystrophy of the gluteal regions, thighs and upper extremities, 69-months after the diagnosis of JDM.

A 14-year-old Caucasian female was admitted with a 6-month history of skin signs and proximal muscle weakness. Clinical features and laboratory tests are listed in Table 1. Combination of prednisone 60 mg (1 mg/kg/day) with subsequent tapering, Chloroquine (CQ) 250 mg QD (later switched to Hydroxychloroquine – HCQ 200 mg BID) along with topical corticosteroids, then Topical Calcineurin Inhibitors (TCI) was initiated. After 6 months, oral Methotrexate (MTX) 15 mg weekly was instituted. Lipodystrophy first appeared in the cheeks and trunk 15 months after JDM diagnosis (21 months from initial JDM symptoms). Despite therapy, after 13 months, partial phenotype progressed to generalized lipodystrophy with the gluteal regions and thighs (28 months after the diagnosis) and upper extremities (69 months after the diagnosis) being affected respectively (Fig. 1). Five years after the diagnosis, JDM relapse occurred, 6 cycles of 3-day Pulse Intravenous Methylprednisolone (IVMP) 500 mg (≈10 mg/kg/day) and oral MTX led to remission without relapse for the next four years, but lipodystrophy persisted.

### Patient 2

**Figure 2:**
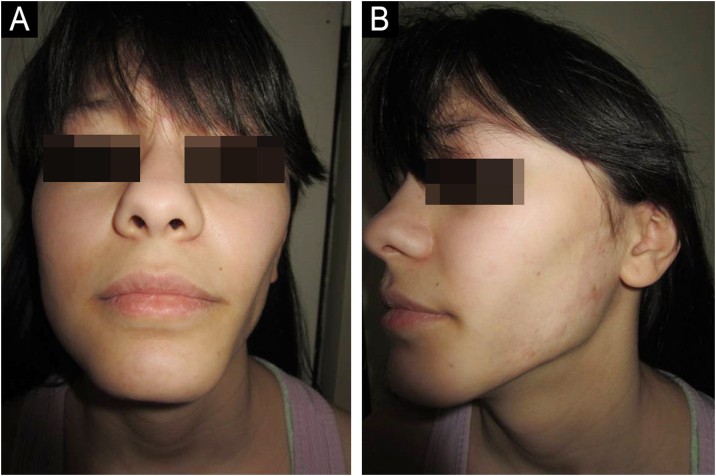
(A‒B) Patient nº 2 with lipodystrophy with subcutaneous atrophy of the left cheek, 48-months after the diagnosis of JDM.

An 8-year-old Caucasian girl was referred with a 2-month history of facial and extremity erythema along with proximal muscle weakness and pain. Prednisone 30 mg daily (1 mg/kg/day, subsequently tapered), and CQ 125 mg/day, led to remission 29-months after the diagnosis. Mild subcutaneous atrophy of the left cheek occurred 18-months after the diagnosis (20-months after initial symptoms) and progressed (Fig. 2). Interestingly, 37-months after the diagnosis and 19-months after the beginning of lipodystrophy, mild calcinosis was noted in the atrophic region. A maintenance dose of CQ 125 mg QD was administered for 9-months in total, and then discontinued. The patient was lesion and symptom-free during the remaining follow-up period, except for lipodystrophy with calcinosis of the left cheek.

### Patient 3

**Figure 3:**
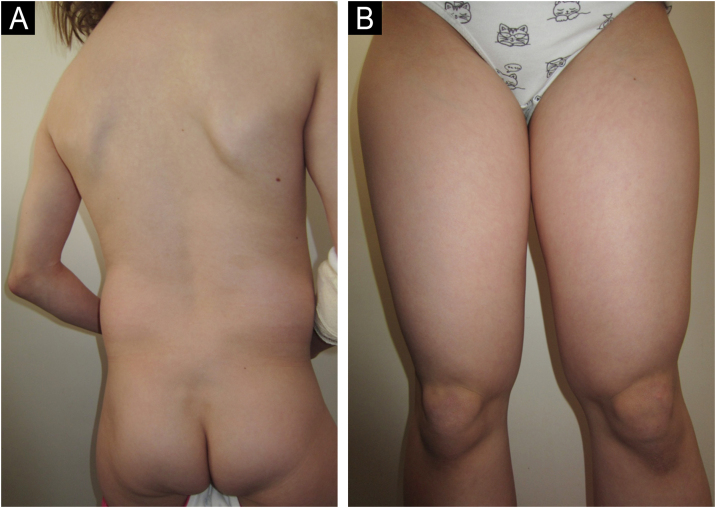
(A‒B) Patient nº 3 with progressive generalized lipodystrophy of the proximal upper extremities, shoulders, gluteal and lumbosacral region, 13-months after the diagnosis of JDM.

**Figure 4:**
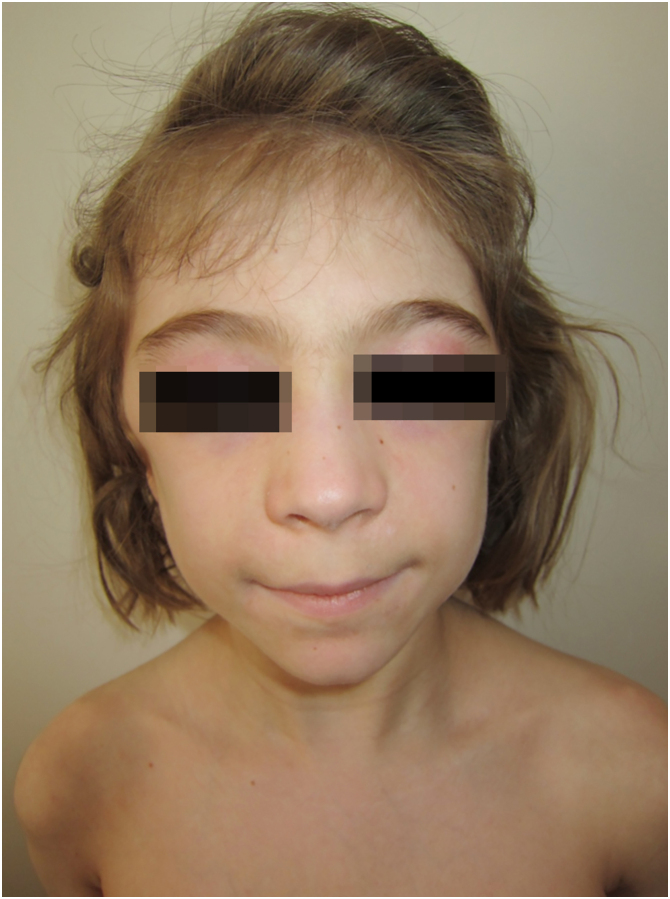
Patient nº 3 with lipodystrophy of the lateral aspects of the face and shoulders, 13 months after the diagnosis of JDM.

An 8-year-old Caucasian girl was admitted with a 20-month history of facial erythema and symmetric muscle weakness. One month after the diagnosis (21-months after initial symptoms) lipodystrophy appeared first on the proximal upper extremities. Nine cycles of 3-day pulse (IVMP) 700 mg (≈30 mg/kg/day), followed by prednisone 50 mg QD (2 mg/kg/day) with subsequent tapering, azathioprine 25 mg TID (3.1 mg/kg/day), and HCQ 100 mg QD (4.1 mg/kg/day) together with TCI were instituted. After 9 months, JDM remission was achieved, but partial lipodystrophy progressed to generalized as the lateral aspects of the face, the gluteal region, the lumbosacral region and the shoulders were also affected (Figure 3, Figure 4). Calcinosis on the left knee was noted 57-months after diagnosis.

### Patient 4

**Figure 5:**
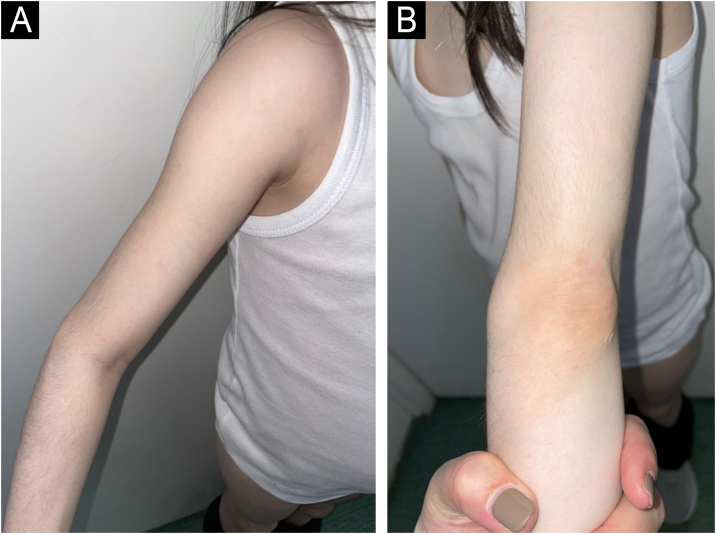
(A‒B) Patient nº 4 with stable lipodystrophy with bilateral atrophy on her upper arms, 16-months after the diagnosis of JDM.

**Figure 6:**
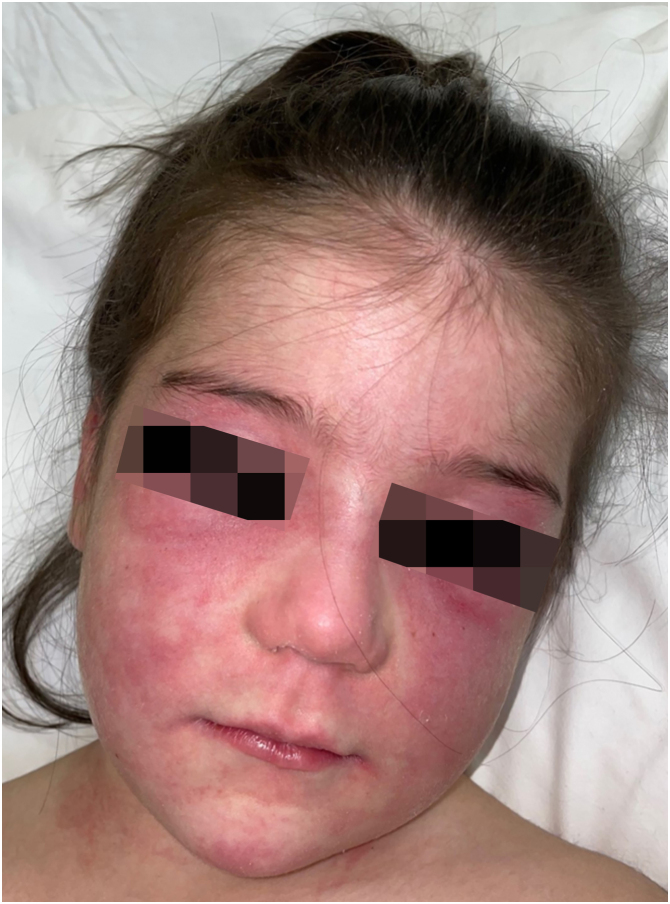
Patient nº 4 with facial erythema, heliotrope sign, and eyelid edema.

A 6-year-old girl was admitted with a 10-month history of facial erythema, arthralgia and generalized muscle weakness. Six months after the first JDM symptoms, bilateral atrophy of the subcutaneous fat tissue on her upper arms appeared and remained stable during follow-up (Figure 5, Figure 6). Twelve cycles of 3-day pulse (IVMP) ranging from 320 mg (≈15 mg/kg/day) to 640 mg (≈30 mg/kg/day) in the last cycle, followed by prednisone 20 mg QD (0.93 mg/kg/day) but increased to 40 mg QD (1.9 mg/kg/day) within one month due to worsening muscle weakness with subsequent tapering, MTX15 mg s.c. weekly, and HCQ 100 mg QD (4.8 mg/kg/day) together with TCI were instituted. After 12-months, muscle strength improved but weakness persisted especially in the lower extremities but laboratory parameters were unremarkable. Calcinosis of the knees and dorsal hands was noted 18 months after the JDM diagnosis. Lipodystrophy of the upper arms persisted during follow-up despite chronic continuous low dose Immunosuppressive Therapy (IST) (Table 1).

### Patient 5

**Figure 7:**
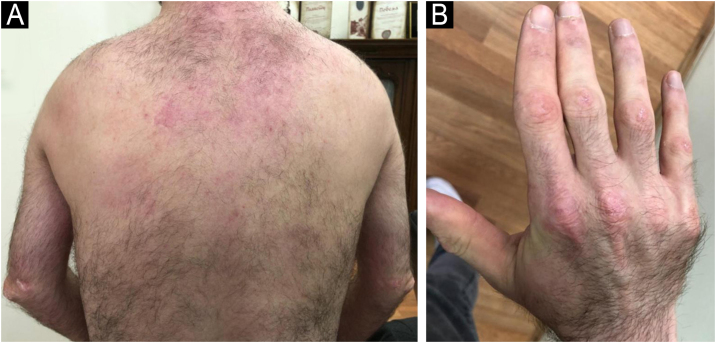
(A‒B) Patient nº 5 lipodystrophy in the shoulder girdle muscles and hands, as well as calcinosis of the right hand and truncal erythema, 290-months after the diagnosis of JDM.

**Figure 8:**
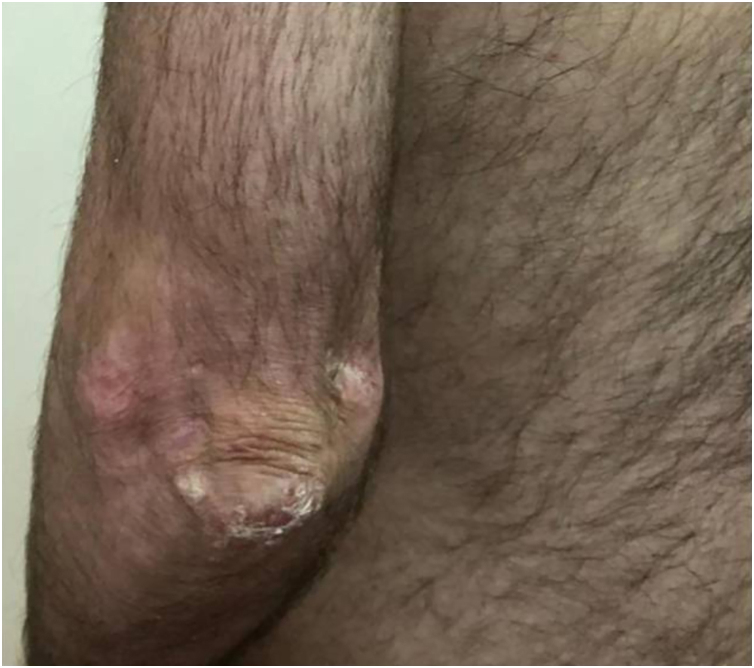
Patient nº 5 with left elbow calcinosis, 290 months after the diagnosis of JDM.

A 3-year-old boy presented with a 6-month history of cutaneous signs, malaise, muscle pain and weakness. A two-year course of IST (oral prednisone 1‒1.5 mg/kg, two cycles of 3-day pulse IVMP 600 mg, two cycles of Intravenous Immunoglobulins (IVIG) and MTX 12.5‒20 mg/weekly) lead to improvement. Calcinosis appeared after almost six years on the forearm and progressed to the left elbow, right knee and hand (after eight years) and gluteus (after nine years). Atrophy of the subcutaneous fat tissue in the shoulder girdle muscles and hands appeared 97 months after diagnosis with no progression during the follow-up. Over the next twenty years, the patient has had multiple episodes of worsening muscle weakness and cutaneous lesions (Figure 7, Figure 8). Maintenance therapy consisted of oral prednisone with varying dosages, systemic antimalarials, and TCI but the patient does not take IST regularly.

## Discussion

Lipodystrophy is a rare complication of JDM and is probably under-reported. JDM onset mostly occurs between 4‒10-years with a 2.7:1 female-to-male ratio in Caucasians.^25^ Similar results were seen in the present study with onset ranging from 3‒14-years and 3.4:1 female-to-male ratio. Misra et al. described 35 patients with childhood ALD, 7 of which had an associated autoimmune disease, but only one JDM.^11^ On the other hand, in a study by Pope et al. of 30 children with childhood ALD, the most frequent underlying diagnosis was JDM with 78%, alone or associated with other autoimmune diseases such as juvenile rheumatoid arthritis in 17%.^10^ JDM differs from other autoimmune diseases such as SLE, which has been reported to occur 4‒37-years after lipodystrophy onset,^26^ while DM usually precedes lipodystrophy onset.^11^, ^12^ Mean time from initial symptoms of JDM to lipodystrophy onset was 34.2-months (Table 1). Overall the time of onset is quite shorter than 3‒4.6-years, as reported in the literature.^10^, ^12^

A study of subcutaneous fat compared 20 JDM patients (of which 40% had lipodystrophy) with controls showed that JDM patients had lower BMI (the difference between controls and patients was 7.6 ± 4.4 kg).^17^ Body Mass Index (BMI) was smaller compared to controls but was not statistically significant.^17^ In the studied patients, the BMI ranged from 14.3–20.7.

The most comprehensive review evaluating clinical and metabolic abnormalities in JDM with lipodystrophy (28 patients) and comparing them to JDM without lipodystrophy was done by Bingham et al.^12^ The analysis determined muscle atrophy, joint contractures, chronic continuous dermatomyositis course, and calcinosis to be the most significant discriminating features when comparing JDM with and without lipodystrophy. Calcinosis, joint contractures, panniculitis and muscle atrophy were found to be predictors for lipodystrophy development in JDM.^12^ In this report, 4/5 (80%) had calcinosis, but in the 3/5 patient’s lipodystrophy preceded calcinosis, while in 1/5 patients calcinosis preceded lipodystrophy by 2 years.

The incidence of calcinosis in JDM is 12%‒70%.^4^, ^5^, ^17^, ^18^, ^19^, ^27^, ^28^ In the present study, the onset of calcinosis after JDM diagnosis ranged from 37‒72-months (mean 46-months).

Published reports have shown IVMP to be effective in shortening rash duration in JDM, decreasing functional impairment and preventing calcinosis development.^27^, ^29^ It is well known that IVMP is inadequate to prevent lipodystrophy and calcinosis if the diagnosis of JDM is delayed.^30^ Huber et al. reported 10/31 JDM patients on IVMP with calcinosis versus 12/34 with calcinosis in the non-IVMP treated group.^5^ Despite the limited number, 4/5 patients who received IVMP had partial or full remission and stabilized muscle enzymes in all patients, but unfortunately, lipodystrophy progressed in two patients despite receiving IVMP and aggressive IST.

In terms of progression, Bingham et al. stated that out of 8 generalized lipodystrophy, only one first presented with partial lipodystrophy, while focal lipodystrophy never progressed to partial or generalized.^12^

Bingham et al. reported^12^ that none of the lipodystrophy patients had MSA which is in line with these findings. All the studied patients were negative for MSA. Unfortunately, the authors did not test anti-p155 antibodies which were reported in 23%‒29% of JDM patients.^31^ Anti-p155 antibody was found more frequently in JDM patients with lipodystrophy compared to JDM without lipodystrophy (6/7 [83%] vs. 27/76 [36%]; p = 0.014), while also more frequent in generalized lipodystrophy compared to partial or focal lipodystrophy.^12^ Furthermore, anti-p140 has been reported in 23% of patients with an association with calcinosis.^32^ In a retrospective review of 96 JDM patients, no significant difference in age, disease activity scores, or lipodystrophy was observed between anti-P155/140 positive JDM, other MSA positive JDM, and MSA negative JDM and trunk:leg fat ratios were similar among the three groups.^33^ One potential mechanism of lipodystrophy is the dysregulation of the complement alternative pathway. Lipodystrophy has been associated with the presence of IgG antibody C3 nephritic factor which utilizes the capacity of the adipocytes to form C3 convertase in its surroundings and induces complement-mediated lysis of adipocytes.^11^ Misra et al. showed that 67% of their patients with acquired partial lipodystrophy had low C3 levels and 83% had C3 nephritic factor.^11^ On the other hand, Bingham et al., reported majority of their patients had normal C3 levels, including all partial lipodystrophy in JDM patients.^12^ This is in line with the analysis, as all our patients had normal C3 levels.

Reports demonstrated that acquired generalized lipodystrophy patients have low leptin levels.^5^, ^34^ Meanwhile in a cross-sectional study of 59 JDM patients, the JDM cohort had higher leptin levels compared to age/sex-matched controls.^35^ Visfatin and apelin-12 were both higher in JDM-active versus JDM-inactive patients and lower adiponectin levels were found in lipodystrophy associated with JDM patients compared to JDM patients without lipodystrophy.^35^ Metabolic dysfunction including insulin resistance, diabetes, hypertriglyceridemia, and non-alcoholic steatohepatitis can be seen. Hypertriglyceridemia has been found to be the first metabolic abnormality in JDM patients, followed by hirsutism and lipodystrophy.^17^ The specificity of hypertriglyceridemia in JDM patients with lipodystrophy is 71%‒100% (12/18, 4/4, 17/24)^7^, ^12^, ^17^ while in JDM patients without lipodystrophy, it has ranged from 17% (9/53)^19^ – 50% (10/20).^17^ Low HDL, high LDL and cholesterol are also associated with JDM and lipodystrophy.^10^, ^11^, ^12^, ^17^, ^35^ This is a stark contrast, as in this study triglycerides were normal in all studied patients. Cholesterol was evaluated in all five patients and elevated only in patient 3. Secondly, insulin resistance and diabetes have been reported as associated complications in JDM, higher percentages in patients with lipodystrophy, especially in generalized and partial forms.^5^, ^7^, ^12^ Based on 20 JDM patients (8 of which had lipodystrophy) a study showed no patients had abnormal OGTT tests.^17^ None of these patients had elevated fasting glucose levels despite corticosteroid therapy. There has been debate about whether the metabolic abnormalities in lipid status and hyperinsulinemia are caused by corticosteroid treatment or the JDM and/or lipodystrophy.^11^ Ilowite et al. showed a difference in characteristic patterns of dyslipoproteinemia due to corticosteroids compared to due to disease activity in pediatric patients with lupus. Corticosteroids resulted in elevated HDL, VLDL, and triglycerides, while disease activity caused low HDL, elevated VLDL and triglycerides.^36^ Therefore, low HDL and elevated triglycerides indicated that dyslipoproteinemia is disease-related.^7^, ^12^

In terms of validated biomarkers for JDM activity, neopterin, CXCL11, and galectin-9 have been considered in perspective.^37^ Furthermore, elevated IFN type I was correlated with skin disease activity and interferon-regulated gene score was significantly higher in JDM than in controls, correlating moderately with JDM activity and strongly with skin activity in anti-TIF1 positive patients.^38^

Metabolic complications in other forms of lipodystrophy have been shown to respond to thiazolidinediones (troglitazone 200‒600 mg daily), causing decreased hemoglobin A1c levels, triglyceride levels, free fatty acid levels and a body fat increase by a mean of 2.4 percentage points.^39^ Lebastchi et al. reported 3 cases of lipodystrophy associated with autoimmune diseases (1 child had JDM), showing 4‒6 year treatment with meterleptin (0.02–0.04 mg/kg s.c.) reduced insulin resistance and hypertriglyceridemia without altering or worsening the autoimmune disease.^34^

All the studied patients had low serum levels of vitamin D at the presentation of JDM (Table 1). The essential role of vitamin D in bone homeostasis is well known, but vitamin D has multiple immunomodulatory effects, including regulation of immune response. Previously it was shown that vitamin D deficiency is associated with disease activity in patients with JDM.^40^ It is important that children and adolescents supplement vitamin D according to current recommendations. More well-designed studies are needed to determine the potential significance of low serum vitamin D levels that the authors found in all patients with lipodystrophy associated with JDM.

## Conclusion

In conclusion, lipodystrophy is a rare, significant, probably under-reported complication of JDM. During the 34-year period, 5/22 (23%) JDM patients developed lipodystrophy. Metabolic complications were not found. All 5 patients had a significant delay in JDM diagnosis. Despite subsequent standard IST, 2/5 patients progressed to generalized lipodystrophy and 4/5 patients developed calcinosis. Early institution of intensive IST is necessary to reduce the risk of lipodystrophy, as a chronic complication of JDM. Further prospective studies using validated measures of outcome are needed for more accurate therapy and long-term outcomes of lipodystrophy associated with JDM.

## Financial support

None declared.

## Authors’ contributions

Igor Kapetanović: Approval of final version; critical literature review; data collection, analysis and interpretation; participation in research orientation; preparation and writing of manuscript; statistical analysis; study conception and planning; manuscript critical review.

Mirjana Gajić-Veljić: Approval of final version; effective participation in research orientation; intellectual participation in therapeutic management; manuscript critical review; conception and planning.

Branka Bonači-Nikolić: Approval of final version; effective participation in research orientation; intellectual participation in therapeutic management; manuscript critical review; study conception and planning; preparation and writing of manuscript.

Miloš Nikolić: Approval of final version; effective participation in research orientation; intellectual participation in therapeutic management; manuscript critical review; study conception and planning.

## Conflicts of interest

None declared.

## References

[1] Mendez E.P., Lipton R., Ramsey-Goldman R., Roettcher P., Bowyer S., Dyer A. (2003). US Incidence of juvenile dermatomyositis, 1995–1998: results from the national institute of arthritis and musculoskeletal and skin diseases registry. Arthritis Rheum..

[2] Bitnum S., Daeschner C.W., Travis L.B., Dodge W.F., Hopps H.C. (1964). Dermatomyositis. J Pediatr..

[3] Huber A., Feldman B.M. (2005). Long-term outcomes in juvenile dermatomyositis: how did we get here and where are we going?. Curr Rheumatol Rep..

[4] Ravelli A., Trail L., Ferrari C., Ruperto N., Pistorio A., Pilkington C. (2010). Long-term outcome and prognostic factors of juvenile dermatomyositis: a multinational, multicenter study of 490 patients. Arthritis Care Res (Hoboken)..

[5] Huber A.M., Lang B., LeBlanc C.M., Birdi N., Bolaria R.K., Malleson P. (2000). Medium- and long-term functional outcomes in a multicenter cohort of children with juvenile dermatomyositis. Arthritis Rheum..

[6] Garg A. (2004). Acquired and inherited lipodystrophies. N Engl J Med..

[7] Huemer C., Kitson H., Malleson P.N., Sanderson S., Huemer M., Cabral D.A. (2001). Lipodystrophy in patients with juvenile dermatomyositis: evaluation of clinical and metabolic abnormalities. J Rheumatol..

[8] Haas N., Henz B.M., Bunikowski R., Keitzer R. (2002). Semicircular lipoatrophy in a child with systemic lupus erythematosus after subcutaneous injections with methotrexate. Pediatr Dermatol..

[9] Eberhard B.A., Ilowite N.T. (2002). Panniculitis and lipodystrophy. Curr Opin Rheumatol..

[10] Pope E., Janson A., Khambalia A., Feldman B. (2006). Childhood acquired lipodystrophy: a retrospective study. J Am Acad Dermatol..

[11] Misra A., Peethambaram A., Garg A. (2004). Clinical features and metabolic and autoimmune derangements in acquired partial lipodystrophy: report of 35-cases and review of the literature. Medicine (Baltimore)..

[12] Bingham A., Mamyrova G., Rother K.I., Oral E., Cochran E., Premkumar A. (2008). Predictors of acquired lipodystrophy in juvenile-onset dermatomyositis and a gradient of severity. Medicine (Baltimore)..

[13] Pretel M., Navedo M., Marques L., Aguado L. (2013). Adult dermatomyositis associated with lipodystrophy. Actas Dermosifiliogr..

[14] Badri T., Ben Hmida M., Benmously-Mlika R., Ben Jennet S., Mokhtar I., Fenniche S. (2013). Focal lipodystrophy without metabolic disorders in adult dermatomyositis. Int J Dermatol..

[15] Le E.N., Abuav R. (2010). Lipodystrophy in association with adult-onset dermatomyositis sine myositis: a rare manifestation. J Am Acad Dermatol..

[16] Lee L.A., Hobbs K.F. (2007). Lipodystrophy and metabolic abnormalities in a case of adult dermatomyositis. J Am Acad Dermatol..

[17] Verma S., Singh S., Bhalla A.K., Khullar M. (2006). Study of subcutaneous fat in children with juvenile dermatomyositis. Arthritis Rheum..

[18] Sharma A., Gupta A., Rawat A., Suri D., Singh S. (2020). Long-term outcome in children with juvenile dermatomyositis: a single-center study from north India. Int J Rheum Dis..

[19] Mathiesen P., Hegaard H., Herlin T., Zak M., Pedersen F.K., Nielsen S. (2012). Long-term outcome in patients with juvenile dermatomyositis: a cross-sectional follow-up study. Scand J Rheumatol..

[20] McCann L.J., Juggins A.D., Maillard S.M., Wedderburn L.R., Davidson J.E., Murray K.J. (2006). The juvenile dermatomyositis national registry and repository (UK and Ireland)-clinical characteristics of children recruited within the first 5 yr. Rheumatology (Oxford)..

[21] Singh S., Bansal A. (2006). Twelve years’ experience of juvenile dermatomyositis in North India. Rheumatol Int..

[22] Misra A., Garg A. (2003). Clinical features and metabolic derangements in acquired generalized lipodystrophy: case reports and review of the literature. Medicine (Baltimore)..

[23] Park J.Y., Javor E.D., Cochran E.K., DePaoli A.M., Gorden P. (2007). Long-term efficacy of leptin replacement in patients with Dunnigan-type familial partial lipodystrophy. Metabolism..

[24] Lundberg I.E., Tjärnlund A., Bottai M., Werth V.P., Pilkington C., Visser M. (2017). 2017 European league against rheumatism/american college of rheumatology classification criteria for adult and juvenile idiopathic inflammatory myopathies and their major subgroups. Ann Rheum Dis..

[25] Cassidy J.T., Petty R.E., Cassidy J.T., Petty R.E. (2001). Textbook of pediatric rheumatology.

[26] Cronin C.C., Higgins T.J., Molloy M. (1995). Lupus. C3 nephritic factor andpartial lipodystrophy. QJM..

[27] Pachman L.M. (1995). Juvenile dermatomyositis: pathophysiology and disease expression. Pediatr Clin North Am..

[28] Rider L.G., Miller F.W. (1997). Classification and treatment of the juvenile idiopathic inflammatory myopathies. Rheum Dis Clin North Am..

[29] Callen A.M., Pachman L.M., Hayford J., Chung A., Ramsey-Goldman R. (1994). Intermittent high-dose intravenous methylprednisolone (IVpulse) therapy prevents calcinosis and shortens disease course in juvenile dermatomyositis. Arthritis Rheum..

[30] Fisler R.E., Liang M.G., Fuhlbrigge R.C., Yalcindag A., Sundel R.P. (2002). Aggressive management of juvenile dermatomyositis results in improved outcome and decreases incidence of calcinosis. J Am Acad Dermatol..

[31] Gunawardena H., Wedderburn L.R., North J., Betteridge Z., Dunphy J., Chinoy H. (2008). Clinical associations of autoantibodies to a p155/140 kDa doublet protein in juvenile dermatomyositis. Rheumatology (Oxford)..

[32] Gunawardena H., Wedderburn L.R., Chinoy H., Betteridge Z.E., North J., Ollier W.E. (2009). Autoantibodies to a 140-kd protein injuvenile dermatomyositis are associated with calcinosis. Arthritis Rheum..

[33] Khojah A., Liu V., Savani S.I., Morgan G., Shore R., Bellm J. (2022). Association of p155/140 autoantibody with loss of nailfold capillaries but not generalized lipodystrophy: a study of ninety-six children with juvenile dermatomyositis. Arthritis Care Res (Hoboken)..

[34] Lebastchi J., Ajluni N., Neidert A., Oral E.A. (2015). A report of three cases with acquired generalized lipodystrophy with distinct autoimmune conditions treated with metreleptin. J Clin Endocrinol Metab..

[35] Marstein H.S., Witczak B.N., Godang K., Olarescu N.C., Schwartz T., Flatø B. (2023). Adipokine profile in long-term juvenile dermatomyositis, and associations with adipose tissue distribution and cardiac function: a cross-sectional study. RMD Open..

[36] Ilowite N.T., Samuel P., Ginzler E., Jacobson M.S. (1988). Dyslipoproteinemia in pediatric systemic lupus erythematosus. Arthritis Rheum..

[37] Khojah A., Morgan G., Pachman L.M. (2021). Clues to disease activity in juvenile dermatomyositis: neopterin and other biomarkers. Diagnostics (Basel)..

[38] Raupov R., Suspitsin E., Preobrazhenskaya E.V., Kostik M. (2024). Interferon type I signature associated with skin disease in juvenile dermatomyositis. Front Med (Lausanne)..

[39] Arioglu E., Duncan-Morin J., Sebring N., Rother K.I., Gottlieb N., Lieberman J. (2000). Efficacy and safety of troglitazone in the treatment of lipodystrophy syndromes. Ann Intern Med..

[40] Robinson A.B., Thierry-Palmer M., Gibson K.L., Rabinovich C.E. (2012). Disease activity, proteinuria, and vitamin D status in children with systemic lupus erythematosus and juvenile dermatomyositis. J Pediatr..

